# Angiotensin II/Angiotensin I Ratio as a New Pharmacodynamic Parameter for Population Modelling in Healthy Adults and Children with Heart Failure Treated with Enalapril

**DOI:** 10.3390/pharmaceutics17101345

**Published:** 2025-10-18

**Authors:** Melina Steichert, Willi Cawello, Bjoern B. Burckhardt, Fabian K. Suessenbach, Stephanie Laeer, on behalf of the LENA Consortium

**Affiliations:** Institute of Clinical Pharmacy and Pharmacotherapy, Heinrich Heine University Düsseldorf, 40225 Düsseldorf, Germany; cawello@hhu.de (W.C.); stephanie.laeer@hhu.de (S.L.)

**Keywords:** enalapril, pharmacokinetic/pharmacodynamic model, angiotensin-converting enzyme inhibitor, angiotensin II/angiotensin I ratio, paediatric, heart failure, dilated cardiomyopathy, congenital heart disease

## Abstract

**Background/Objectives**: Since 2023, enalapril orodispersible minitablets have been approved in Europe for paediatric heart failure from birth, but no population pharmacodynamic analyses have yet been conducted in this patient group. The objectives were to investigate the effect of the active metabolite enalaprilat on the angiotensin II/angiotensin I ratio in children with heart failure and to determine potential differences compared to healthy adults. **Methods**: Population pharmacokinetic/pharmacodynamic analysis for healthy adults and population pharmacodynamic analysis for children with heart failure were performed using Monolix. Data were analyzed from 9 healthy adults after a single dose of enalapril and from 27 angiotensin-converting enzyme (ACE) inhibitor-naïve children with heart failure treated with enalapril orodispersible minitablets for up to one year in the ‘Labeling of Enalapril from Neonates up to Adolescents’ (LENA) studies. **Results**: For the relationship between enalaprilat and the angiotensin II/angiotensin I ratio, a maximum inhibition (I_max_) model with full inhibition and sigmoidicity factor was selected for healthy adults and without sigmoidicity factor for children with heart failure. In children with heart failure, the population estimate for the baseline effect was higher (0.19 versus 0.043) and for the half-maximal inhibitory concentration lower (1.19 µg/L versus 30.01 µg/L) than in healthy adults. Four hours after a median initial dose of 0.06 mg/kg enalapril maleate, the angiotensin II/angiotensin I ratio decreased by a median of 79.3% in children with heart failure. **Conclusions**: Effective ACE inhibition can be assumed at the administered dose in children with heart failure. Population analyses suggest that children with heart failure may be more sensitive to enalaprilat than healthy adults.

## 1. Introduction

Recent studies have shown clinical improvements in children with heart failure treated with the angiotensin-converting enzyme (ACE) inhibitor enalapril. In the ‘Labeling of Enalapril from Neonates up to Adolescents’ (LENA) studies, children with heart failure due to congenital heart disease or dilated cardiomyopathy were treated with enalapril orodispersible minitablets for up to one year [1]. An evaluation of children with congenital heart disease who participated in the LENA project showed that after 8 weeks of treatment with enalapril, the severity of heart failure score, the left ventricular diastolic dimension z-score, and N-terminal pro-B-type natriuretic peptide (NT-proBNP) levels were significantly reduced [2]. In a 52-week randomized, double-blind clinical efficacy study of sacubitril/valsartan versus enalapril in children aged 1 month to less than 18 years with heart failure due to systemic left ventricular systolic dysfunction, the superiority of sacubitril/valsartan could not be demonstrated [3,4]. With enalapril, as with sacubitril/valsartan, a clinically meaningful reduction in heart failure severity score and NT-proBNP was observed in the study. For both enalapril and sacubitril/valsartan, age-appropriate dosage forms for the treatment of paediatric heart failure were approved in Europe in 2023 [5,6]. Unlike sacubitril/valsartan granules in capsules for opening, which are approved for children aged one year and older, enalapril orodispersible minitablets are already approved for use from birth. In view of the positive study results and the age-appropriate dosage form approved for use from birth, it is expected that the use of enalapril in children with heart failure will increase.

The pharmacokinetics of the active metabolite enalaprilat in children with heart failure are influenced by age and heart failure. Enalaprilat is a potent ACE inhibitor but is poorly absorbed after oral administration [7]. Therefore, the inactive prodrug enalapril is used, which has better oral bioavailability and is hydrolysed to enalaprilat by hepatic carboxylesterase 1 [7]. Previously, a simultaneous population pharmacokinetic analysis of enalapril and enalaprilat in ACE inhibitor-naïve children with heart failure from the LENA project was conducted [8]. The population pharmacokinetic analysis showed an increase in weight-adjusted apparent clearance of enalaprilat with increasing age. Furthermore, it was shown that with increasing Ross score, a measure of the severity of heart failure, the weight-adjusted apparent volume of distribution of enalaprilat decreases.

Age and heart failure might affect the pharmacodynamics of the active metabolite enalaprilat in children with heart failure. Systematic reviews revealed an age-related decline in plasma renin activity, angiotensin I, angiotensin II, and aldosterone in healthy children during childhood [9,10,11]. In addition, the renin–angiotensin–aldosterone system is activated as a compensatory mechanism in children with heart failure [12]. Since age and heart failure influence the parameters of the renin–angiotensin–aldosterone system, the question arises whether the pharmacodynamics of enalaprilat differ in children with heart failure compared to healthy adults.

Model-dependent pharmacodynamic analyses in adults used various pharmacodynamic parameters. The effect of the active metabolite enalaprilat on ACE activity or blood pressure in healthy adults and adults with hypertension was investigated using pharmacokinetic/pharmacodynamic modelling [13,14,15,16,17]. In addition, two physiologically based pharmacokinetic/pharmacodynamic models and one population pharmacokinetic/pharmacodynamic model were developed, describing the effect of enalaprilat on the individual parameters of the renin–angiotensin–aldosterone system in adults [18,19,20].

Model-dependent pharmacodynamic analyses for enalaprilat based on paediatric data are limited. Kechagia et al. [21] developed a pharmacokinetic/pharmacodynamic model using pharmacokinetic data from children and blood pressure data from adults with hypertension obtained from the literature. The pharmacokinetic/pharmacodynamic model was then validated using blood pressure data from children with hypertension obtained from the literature.

The population approach using nonlinear mixed effects modelling has the advantage that even sparse and unbalanced data sets, as frequently found in children, can be analyzed through the simultaneous analysis of all data [22]. The input data for a population pharmacokinetic/pharmacodynamic model consists of pharmacokinetic and pharmacodynamic observations, time, dose, and, if applicable, covariates of the subjects. As a result, estimates are obtained for fixed effect parameters such as clearance for pharmacokinetics and half-maximal inhibitory concentration for pharmacodynamics, as well as estimates for random effects, including interindividual and residual variability [22].

Model-dependent population analyses in children with heart failure using suitable pharmacodynamic parameters could help to gain insights into the pharmacodynamic effects of enalaprilat in this population and possible differences compared to adults. Blood pressure is less suitable as a pharmacodynamic parameter in children with heart failure, as it was observed that blood pressure did not change significantly during the study period in children with congenital heart disease who were treated with enalapril and participated in the LENA project [2]. Unlike blood pressure, ACE activity has the advantage that it is directly influenced by enalaprilat without any intermediate steps. As ACE activity is not always available, alternative pharmacodynamic parameters may be useful.

**Figure 1:**
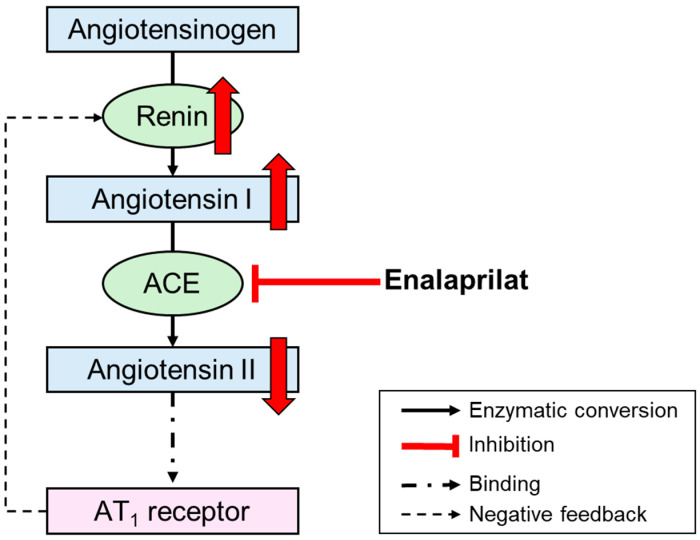
Schematic diagram of the renin–angiotensin–aldosterone system and illustration of the effects of enalaprilat on renin, angiotensin I, and angiotensin II. The red arrows indicate the changes in pharmacodynamic parameters caused by the inhibition of angiotensin-converting enzyme by enalaprilat. Angiotensin II decreases, leading to reduced negative feedback on renin secretion. This results in an increase in renin, which in turn leads to an increase in angiotensin I. ACE, angiotensin-converting enzyme; AT_1_ receptor, angiotensin II type 1 receptor.

Since the angiotensin II/angiotensin I ratio is influenced by the administration of enalapril, it could serve as a pharmacodynamic parameter. The active metabolite enalaprilat inhibits the ACE, which normally converts angiotensin I to angiotensin II (Figure 1). After administration of enalapril to healthy adults, angiotensin I levels increased significantly, and angiotensin II levels decreased significantly [23]. Therefore, the angiotensin II/angiotensin I ratio was used as a measure of the in vivo ACE activity in healthy adults [24,25]. A previous model-independent analysis in ACE inhibitor-naïve children with heart failure from the LENA project showed a significant increase in plasma renin activity at the dose confirmation visit, which took place on average 5 days after the first dose of enalapril [9]. Since renin converts angiotensinogen into angiotensin I, it is likely that the children with heart failure also showed an increase in angiotensin I after administration of enalapril. In another study in children with congestive heart failure, angiotensin II levels decreased significantly after administration of enalapril, and plasma renin activity also increased significantly [26]. Consequently, it can be assumed that the angiotensin II/angiotensin I ratio can also serve as a measure of in vivo ACE activity in children with heart failure. To the authors’ knowledge, there are no model-dependent population analyses that use angiotensin II/angiotensin I as a pharmacodynamic parameter for healthy adults or children with heart failure.

The objectives were to gain insights into the effect of the active metabolite enalaprilat on the angiotensin II/angiotensin I ratio in children with heart failure and to identify possible differences compared to healthy adults. For this purpose, a population pharmacokinetic/pharmacodynamic model was developed that describes the effect of the active metabolite enalaprilat on the angiotensin II/angiotensin I ratio in healthy adults for whom extensive data were available. The predictions of the pharmacokinetic/pharmacodynamic model for adults were used to compare the angiotensin II/angiotensin I ratio after administration of enalapril in healthy adults and children with heart failure. In addition, a population pharmacodynamic model was developed based on simultaneous pharmacokinetic and pharmacodynamic measurements in ACE inhibitor-naïve children with heart failure from the LENA project. The previous population analysis in this population examined the pharmacokinetics of enalapril and enalaprilat, as well as clinically relevant covariates for dosing [8]. This study focuses on the pharmacodynamics of enalaprilat in ACE inhibitor-naïve children with heart failure.

## 2. Materials and Methods

### 2.1. Adult Data

#### 2.1.1. Study Design and Investigated Population

The adult data used were obtained from an open-label, single-sequence, single-dose study in nine healthy subjects [27]. The enalaprilat, angiotensin I, and angiotensin II measurements from this study were analyzed. The study was conducted in 2013 at the Institute of Clinical Pharmacy and Pharmacotherapy at Heinrich Heine University Düsseldorf in Germany according to the Declaration of Helsinki and Good Clinical Practice recommendations. The Ethics Committee of the Medical Faculty of the Heinrich Heine University Düsseldorf granted the ethical approval (proposal no. 3809 [including all amendments]). The study was registered in the German Clinical Trials Register (DRKS00037310). Prior to participation, all subjects provided written informed consent.

Subjects were included in the study if they were at least 18 years old, had a body weight of more than 50 kg, and were free from any known organ diseases. Exclusion criteria were angioedema, urticaria, known allergies, or low blood pressure values in the past (<90/60 mm Hg), as well as pregnancy in female subjects.

#### 2.1.2. Dosing

After a fasting period of at least 10 h, one tablet of 20 mg enalapril maleate (EnaHEXAL^®^, HEXAL, Holzkirchen, Germany) was administered to the subjects with 250 mL of water. The intake of xanthine-containing food or beverages and alcohol was prohibited for at least 48 h after administration.

#### 2.1.3. Sampling

The time points for pharmacokinetic sampling on the first study day were predose, every 10 min for the first 3 h after administration, every 20 min for the next 3 h and thereafter every 30 min up to 8 h after administration. Additional pharmacokinetic samples were collected 24, 48 and 72 h after administration.

The time points for pharmacodynamic sampling were predose, 0.5, 1, 2, 3, 4, 5, 6, 7, 8, 24, 48, and 72 h after administration.

On the first day of the study, blood samples were taken through an indwelling cannula in a peripheral arm vein. The latter blood samples were taken through direct venipuncture. All blood samples were drawn after the participants had been lying in a supine position for at least 30 min. The materials used and the sample preparation have been described elsewhere [27].

#### 2.1.4. Analytical Methods for Samples from Healthy Adults

Pharmacokinetic serum samples (enalaprilat) were purified by solid-phase extraction and analyzed using liquid chromatography–triple quadrupole tandem mass spectrometry (Shimadzu HPLC 10 [Shimadzu, Duisburg, Germany] coupled with AB Sciex API 2000 mass spectrometer [Sciex, Darmstadt, Germany]). The lower limit of quantification (LLOQ) of enalaprilat was 0.70 µg/L.

Angiotensin I was determined using a commercial ^125^I radioimmunoassay (Immunotech, Prague, Czech Republic) and had a calibration range of 0.2 to 30 µg/L. For the determination of angiotensin II, a solid-phase extraction was combined with a modified enzyme-linked immunosorbent assay (IBL, Hamburg, Germany). The calibration range of angiotensin II was between 1 and 125 pg/mL. Further information regarding the analytical methods can be found elsewhere [27].

### 2.2. Paediatric Data

#### 2.2.1. Study Design and Investigated Population

The sources for the paediatric data were two multicentre, prospective, open-label, phase II/III pharmacokinetic bridging studies and a multicentre, prospective, open-label, phase II/III safety follow-up study of the LENA project [1]. The studies were carried out from 2016 to 2018 in hospitals in Austria, Germany, Hungary, The Netherlands (2 sites), and Serbia (2 sites). In the safety follow-up study, there were no subjects at the study centre in Germany. The ethics committees of the participating institutions approved the studies. Prior to the inclusion of each subject in the studies, informed parental consent was obtained. According to national requirements, the assent of the participating children was obtained. The studies were registered on the EU Clinical Trials Register (EudraCT 2015-002335-17, EudraCT 2015-002396-18 and EudraCT 2015-002397-21).

In the pharmacokinetic bridging studies, children with heart failure due to dilated cardiomyopathy (EudraCT 2015-002335-17) or congenital heart disease (EudraCT 2015-002396-18) were treated with enalapril orodispersible minitablets for 8 weeks. Part of the pharmacokinetic bridging studies was also an exploratory pharmacodynamic assessment. Subjects with and without prior ACE inhibitor treatment were included. In the present work, the data of the children without ACE inhibitor pretreatment were analyzed; therefore, all the following information refers to ACE inhibitor-naïve children. Male and female patients weighing more than 2.5 kg with heart failure due to congenital heart disease or dilated cardiomyopathy were included. Inclusion criteria for subjects with congenital heart disease were that they required after load reduction by drug therapy and were between birth and under 6 years of age. Subjects with dilated cardiomyopathy were included if they had left ventricular end-diastolic dimension > P95 and/or left ventricular shortening fraction < 25%. The age of subjects with dilated cardiomyopathy had to be between 1 month and 12 years old. A list of exclusion criteria for the pharmacokinetic bridging studies can be found in the Supplementary Materials (Table S1).

In the safety follow-up study (EudraCT 2015-002397-21), subjects from the pharmacokinetic bridging studies who were still treated with enalapril orodispersible minitablets or who were treated with enalapril orodispersible minitablets for at least 3 days were included. There were no additional exclusion criteria in the safety follow-up study. During the 10-month study period, pharmacodynamic sampling was performed in all subjects and pharmacokinetic sampling in those subjects who were still receiving enalapril orodispersible minitablets.

#### 2.2.2. Dosing

For the studies, a dosing regimen with age- and weight-dependent recommended titration doses, target doses, and maximum doses was developed using physiologically based pharmacokinetic simulation (Table S2). The appropriate dosage for each subject was selected based on the judgment of the investigator. Daily doses ≤ 0.25 mg were administered once daily in the morning, and daily doses > 0.25 mg were administered in two equal divided doses in the morning and evening. The required number of orodispersible minitablets with 0.25 mg enalapril maleate or 1 mg enalapril maleate (now approved as Aqumeldi^®^, Proveca Pharma Limited, Dublin, Ireland) was placed in the patient’s cheek pouch. The orodispersible minitablets rapidly disintegrated into small, easily swallowable particles. To facilitate swallowing, a fluid of the patient’s/parent’s choice (e.g., breast milk, formula milk, cow milk, water) could be administered.

The parents documented the time points of orodispersible minitablet administration in a patient diary for at least seven days before each study visit. The investigator or study nurse transferred the dosing times of the seven days prior to the study visit from the patient diary to electronic case report forms. In addition, dosing time on the study visit was noted in the electronic case report forms.

#### 2.2.3. Sampling

In the pharmacokinetic bridging studies, a pharmacokinetic/pharmacodynamic profile was usually obtained in ACE inhibitor-naïve subjects at the initial dose visit, with pharmacokinetic samples collected before and 1, 2, 4, 6, and 12 h after enalapril orodispersible minitablet administration, as well as pharmacodynamic samples collected before and 4 h after administration. Alternatively, the pharmacokinetic/pharmacodynamic profile could also be obtained after reaching steady state at the optimal dose. In this case, the 12 h pharmacokinetic sample was omitted. Single pharmacokinetic and pharmacodynamic samples were collected in immediate succession during the remaining study visits. These samples were collected predose during titration and at the end of the studies. For all other visits, the investigator could determine the time of sampling. When the treatment with enalapril orodispersible minitablets was discontinued, no further pharmacokinetic samples were taken from the subjects concerned.

During the four study visits of the safety follow-up study, single pharmacokinetic samples were taken from subjects still treated with enalapril orodispersible minitablets, and single pharmacodynamic samples were taken from all subjects. For these samples, the investigator could determine the time of sampling. In subjects from whom pharmacokinetic and pharmacodynamic samples were taken, these samples were collected in immediate succession.

The predefined time points of the study visits are listed in the Supplementary Materials (Table S3). The exact sampling times were noted in the electronic case report forms for all pharmacokinetic and pharmacodynamic samples. The subjects were in a supine position for blood sampling. The materials used and the preparation of the pharmacokinetic samples can be found elsewhere [28].

For the collection of the analyzed pharmacodynamic samples, ethylenediaminetetraacetic acid (EDTA) Monovettes^®^ (Sarstedt, Nuembrecht, Germany) were used, which were spiked with an inhibitor cocktail [dimethyl sulfoxide (AppliChem GmbH, Darmstadt, Germany), 1,10-phenanthroline (Sigma-Aldrich, Steinheim, Germany), pepstatin A (Sigma-Aldrich, Steinheim, Germany), 4-hydroxymercuri benzoic acid (Sigma-Aldrich, Steinheim, Germany), and EDTA (Carl Roth GmbH + Co. KG, Karlsruhe, Germany)] to block degradation of humoral parameters. In situations where sampling with EDTA Monovettes^®^ was not possible, micro collection tubes containing EDTA solution and the aforementioned inhibitor cocktail were used. The blood collection tubes were stored on ice before, during, and after blood collection. Immediately afterwards, the blood collection tubes were centrifuged for 10 min at 2000× *g* under cooled conditions (0–4 °C). The transferred supernatant was snap-frozen and stored at −80 °C until analysis.

#### 2.2.4. Analytical Methods for Paediatric Samples

After solid-phase extraction, pharmacokinetic serum samples (enalaprilat) were analyzed via liquid chromatography–triple quadrupole tandem mass spectrometry (Shimadzu HPLC 10 [Shimadzu, Duisburg, Germany] coupled with AB Sciex API 2000 mass spectrometer [Sciex, Darmstadt, Germany]). The calibration range for enalaprilat was 0.18 to 180 µg/L. The analytical methods used for the paediatric pharmacokinetic samples have been described in more detail elsewhere [28].

For the simultaneous determination of several angiotensin peptides, including angiotensin I and angiotensin II, a validated multiplex liquid chromatography high-resolution mass spectrometry method was applied [29]. Following precipitation and solid-phase extraction, the samples were analyzed using a Nexera XR liquid chromatography system (Shimadzu, Duisburg, Germany) coupled to a TripleTOF^®^ 6600 mass spectrometer from AB Sciex (Concord, ON, Canada). The calibration range was 25.4 to 1594.2 pg/mL for angiotensin I and 22.3 to 1395.8 pg/mL for angiotensin II.

### 2.3. Software

The population pharmacokinetic/pharmacodynamic and population pharmacodynamic analyses were conducted using the nonlinear mixed effects modelling program Monolix version 2024R1 (Lixoft, Antony, France). For the estimation of the population parameters, Monolix uses a stochastic approximation expectation-maximization algorithm. Excel^®^ version 2406 (Microsoft, Redmond, WA, USA) and R version 4.2.2 (The R Foundation for Statistical Computing, Vienna, Austria) were used to generate the input files for Monolix, statistical analyses and additional graphics.

### 2.4. Population Pharmacokinetic/Pharmacodynamic Modelling for Healthy Adults

#### 2.4.1. Model Development

First, the angiotensin II/angiotensin I ratio was plotted against the enalaprilat concentration for each subject to check for hysteresis. Thereafter, a pharmacokinetic model for the active metabolite enalaprilat was developed. A one- and two-compartment model with first-order absorption and linear elimination was tested for enalaprilat. In addition, the implementation of a lag time as well as transit compartments was tested. A combined residual error model with an additive and a proportional error was selected as the error model. The conditional sampling use for stepwise approach based on correlation tests (COSSAC) method was used for the covariate search [30]. The potential covariates sex, age, weight, and body mass index were tested as covariates for the model parameters with random effects. An exponential model was tested for the categorical covariate sex, and a power model scaled with the weighted mean of the respective covariates was tested for the continuous covariates age, weight, and body mass index. For the likelihood ratio threshold, the default setting *p* = 0.01 was used as the forward and backward threshold. Based on the scatter plots for each pair of random effects and the results of the correlation test using a *t*-test, the inclusion of potential correlations between random effects in the model was tested.

The pharmacokinetic model developed was then used for the pharmacokinetic part of the pharmacokinetic/pharmacodynamic model, with the parameter estimates of the pharmacokinetic model serving as initial estimates. For pharmacokinetic/pharmacodynamic modelling, the simultaneous approach was chosen, in which all model parameters are estimated together. A maximum inhibition (I_max_) model with an effect compartment was selected for the pharmacodynamic part of the model. For the I_max_ model, partial inhibition and full inhibition were tested, as well as the addition of a sigmoidicity factor. For the pharmacodynamic part of the model, a proportional error model and a combined error model with an additive and a proportional error were tested as the residual error model. As described above, a covariate search was then performed, and the inclusion of correlations between random effects was tested.

Interindividual variability was incorporated exponentially for the parameters, as it was assumed that the interindividual variability of the parameters is lognormally distributed. For model selection, the change in the objective function, the change in the residual and interindividual variability, the change in the relative standard errors of the parameter estimates, and the visual inspection of the goodness-of-fit plots were considered. Bioavailability could not be determined based on the available data; therefore, the apparent clearance, the apparent intercompartmental clearance, the apparent central volume of distribution and the apparent peripheral volume of distribution are reported. Predose pharmacokinetic samples were excluded because no concentration was expected at this time in healthy adults without prior treatment with enalapril. Pharmacokinetic samples with concentrations below the limit of quantification were treated as censored, and a lower limit of zero was set (equivalent to the M4 method in NONMEM^®^) [31]. For pharmacodynamic samples with concentrations below the limit of quantification, the reported value was used to calculate the angiotensin II/angiotensin I ratio.

#### 2.4.2. Model Evaluation

To evaluate the final pharmacokinetic/pharmacodynamic model, the goodness-of-fit plots and visual predictive checks were visually inspected. For the visual predictive checks, 500 Monte Carlo simulations were performed with the final pharmacokinetic/pharmacodynamic model and the design structure of the original dataset.

### 2.5. Comparison of the Time Course of the Effect in Healthy Adults and Children with Heart Failure

To compare the time course of the angiotensin II/angiotensin I ratio after enalapril administration in healthy adults and children with heart failure, the corresponding visual predictive check of the final pharmacokinetic/pharmacodynamic model for healthy adults was used. The observed data from children with heart failure were added to the visual predictive check. In contrast to the adult data, data were also available for children with heart failure after repeated administration of enalapril. For this data, the time since the last dose was calculated. If the angiotensin II value was below the LLOQ, the angiotensin II value was replaced by LLOQ/2 before calculating the angiotensin II/angiotensin I ratio. Observations in which both angiotensin I and angiotensin II were below the LLOQ were excluded.

### 2.6. Change in the Angiotensin II/Angiotensin I Ratio After Initial Enalapril Dose in Children with Heart Failure

The percentage change in the angiotensin II/angiotensin I ratio was calculated in children with heart failure in whom an angiotensin II/angiotensin I ratio was available before the first dose of enalapril and four hours afterwards.

### 2.7. Population Pharmacodynamic Modelling for Children with Heart Failure

#### 2.7.1. Model Development

The angiotensin II/angiotensin I ratio and the simultaneously measured enalaprilat concentrations were used as input data for the pharmacodynamic model. An I_max_ model was selected as the pharmacodynamic model. Analogous to the adult model, partial inhibition and full inhibition were tested, as well as the addition of a sigmoidicity factor. A proportional error model and a combined error model with an additive and a proportional error were investigated to describe residual variability.

Exponential models were used for the interindividual variability of the parameters, as a lognormal distribution of the interindividual variability of the parameters was assumed. For the model selection, the same points were considered as for the adult model. The angiotensin II value was substituted by LLOQ/2 for the calculation of the angiotensin II/angiotensin I ratio if the angiotensin II value was below the LLOQ. Observations were excluded if angiotensin I and angiotensin II were below the LLOQ.

#### 2.7.2. Model Evaluation

The final pharmacodynamic model was evaluated by visual inspection of the goodness-of-fit plots and the visual predictive check. A total of 500 Monte Carlo simulations using the final pharmacodynamic model and the design structure of the original dataset were performed for the visual predictive check.

## 3. Results

### 3.1. Adult Data

A total of 288 serum enalaprilat concentrations from 9 healthy subjects were included in the analysis. Of these, 47 (16.3%) concentrations were below the LLOQ and were treated as censored, with the lower limit set at zero (M4 method). In 2 subjects, 9 of the 34 scheduled pharmacokinetic samples were not available. Additionally, pharmacokinetic samples taken prior to administration of enalapril (n = 9) were excluded because no concentration was expected at that time in healthy adults who had not previously been treated with enalapril.

In total, 99 calculated angiotensin II/angiotensin I ratios from the 9 healthy subjects were included in the analysis. The angiotensin II/angiotensin I ratios 48 and 72 h after administration of enalapril were excluded because the angiotensin II/angiotensin I ratio rose above the baseline effect in some subjects. Furthermore, the maximum dosage interval for enalapril therapy in patients is 24 h. Five (5.1%) angiotensin I values and one (1.0%) angiotensin II value were below the LLOQ. Due to the low number of values below the LLOQ, the reported values were used to calculate the angiotensin II/angiotensin I ratio. The patient characteristics of the nine healthy subjects are summarised in Table 1.

### 3.2. Paediatric Data

Overall, 54 simultaneous serum concentrations of enalaprilat and angiotensin II/angiotensin I ratios from 27 children with heart failure were included. The angiotensin II value was replaced by LLOQ/2 in 55.6% of the cases because the angiotensin II value was below the LLOQ. Of the enalaprilat serum concentrations, only the measurements prior to the first dose of enalapril were below the LLOQ and were set to zero. Measurements were excluded from analysis because angiotensin I was above the calibration range (n = 10), enalapril therapy was discontinued or interrupted (n = 10), the timepoint of discontinuation of enalapril therapy was unclear (n = 8), angiotensin I and angiotensin II were below the LLOQ (n = 2), the time since the last dose was more than 24 h (n = 1), or other uncertainties existed (n = 2). The included observations could be divided into observations prior to the first dose of enalapril (n = 16), observations after the first dose of enalapril (n = 12), and observations after repeated doses of enalapril (n = 26). Observations after repeated doses of enalapril ranged from 3.9 days to 11.8 months after the first dose of enalapril. Two subjects did not receive their enalapril orodispersible minitablets on the evening before the measurement. The patient characteristics of the 27 children with heart failure are given in Table 1.

**Table 1:** Characteristics of the healthy adults and children with heart failure.

Characteristic	Number of Observations (%)	Mean (SD)	Median (Range)
Healthy adults (n = 9)			
Age (years)	9 (100)	22.7 (4.0)	21 (19–30)
Weight (kg)	9 (100)	69.8 (13.6)	74 (47–88)
Body mass index (kg/m^2^)	9 (100)	21.3 (2.4)	22 (18–25)
Sex			
Male	6 (66.7)	-	-
Female	3 (33.3)	-	-
Children with heart failure (n = 27)			
Age (years)	54 (100)	0.49 (0.47)	0.36 (0.07–2.24)
Weight (kg)	54 (100)	5.5 (2.2)	4.8 (3.2–13.0)
Ross score	54 (100)	4.0 (2.6)	4 (0–9)
Sex			
Male	12 (44.4)	-	-
Female	15 (55.6)	-	-
Aetiology of heart failure			
Dilated cardiomyopathy	3 (11.1)	-	-
Congenital heart disease	24 (88.9)	-	-

SD, standard deviation.

### 3.3. Population Pharmacokinetic/Pharmacodynamic Modelling for Healthy Adults

#### 3.3.1. Population Pharmacokinetic/Pharmacodynamic Model

**Figure 2:**
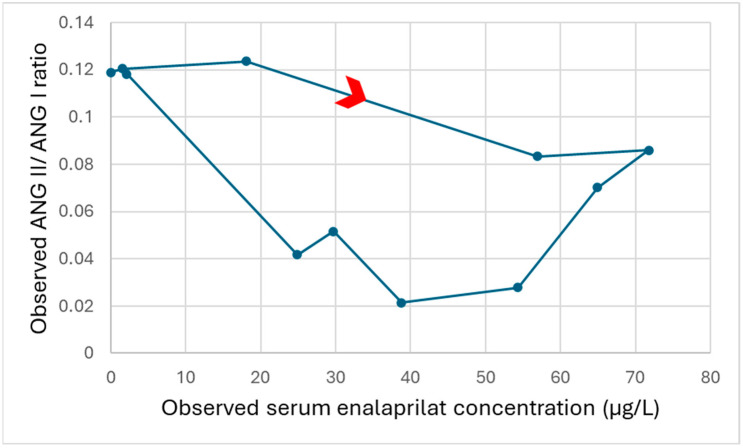
Effect concentration curve of a healthy adult subject. The points in the plot are connected in chronological order of measurement. The first measurement was the one with an observed serum enalaprilat concentration of zero. The red arrow indicates the direction of the chronological order. ANG I, angiotensin I; ANG II, angiotensin II.

The diagrams showing the angiotensin II/angiotensin I ratio versus the enalaprilat concentration revealed varying degrees of clockwise hysteresis in the individual subjects. Exemplary, Figure 2 shows the effect concentration curve of one healthy adult subject, with the data points connected in chronological order of measurement. A clockwise hysteresis can be seen, as a given concentration at a later measurement time point leads to a lower angiotensin II/angiotensin I ratio. Since a greater reduction in the angiotensin II/angiotensin I ratio represents a stronger effect, the clockwise hysteresis shows that the effect increased with time at a given concentration. An effect compartment was integrated into the pharmacokinetic/pharmacodynamic model because pronounced clockwise hysteresis was observed in some subjects.

For the pharmacokinetic part of the pharmacokinetic/pharmacodynamic model, a two-compartment model with first-order absorption and linear elimination with transit compartments was selected based on the predefined model selection criteria. In contrast to the one-compartment model, the two-compartment model captured the observed biphasic exponential decline in enalaprilat concentrations. The delayed increase in enalaprilat concentration was better captured with transit compartments than with a lag time. The delay is due to the time required for enalapril to be absorbed and converted to enalaprilat. In this model, the absorption rate constant therefore considers the absorption of enalapril and its conversion to enalaprilat.

For the pharmacodynamic part of the pharmacokinetic/pharmacodynamic model, an I_max_ model with full inhibition and a sigmoidicity factor was selected in accordance with the predefined model selection criteria. Full inhibition was chosen, as the model with partial inhibition estimated an I_max_ of 99% for all subjects. Adding the sigmoidicity factor contributed to achieving a relative standard error of less than 40% for all parameters.

A combined residual error model with an additive and a proportional error was used to describe the residual variability of the pharmacokinetic, while a proportional error model was used to describe the residual variability of the pharmacodynamic. Since the predefined thresholds were not reached, no covariate relationship was included in the model. A positive correlation between the random effects of the apparent central volume of distribution of enalaprilat and the apparent clearance of enalaprilat was identified and included in the model. Interindividual variability for the absorption rate constant, the apparent intercompartmental clearance of enalaprilat, the apparent peripheral volume of distribution of enalaprilat, the effect compartment transfer rate constant, and the sigmoidicity factor was removed because the data were not sufficient to leave interindividual variability for all parameters in the model. The parameter estimates of the final pharmacokinetic/pharmacodynamic model for healthy adults are shown in Table 2.

#### 3.3.2. Model Evaluation

**Figure 3:**
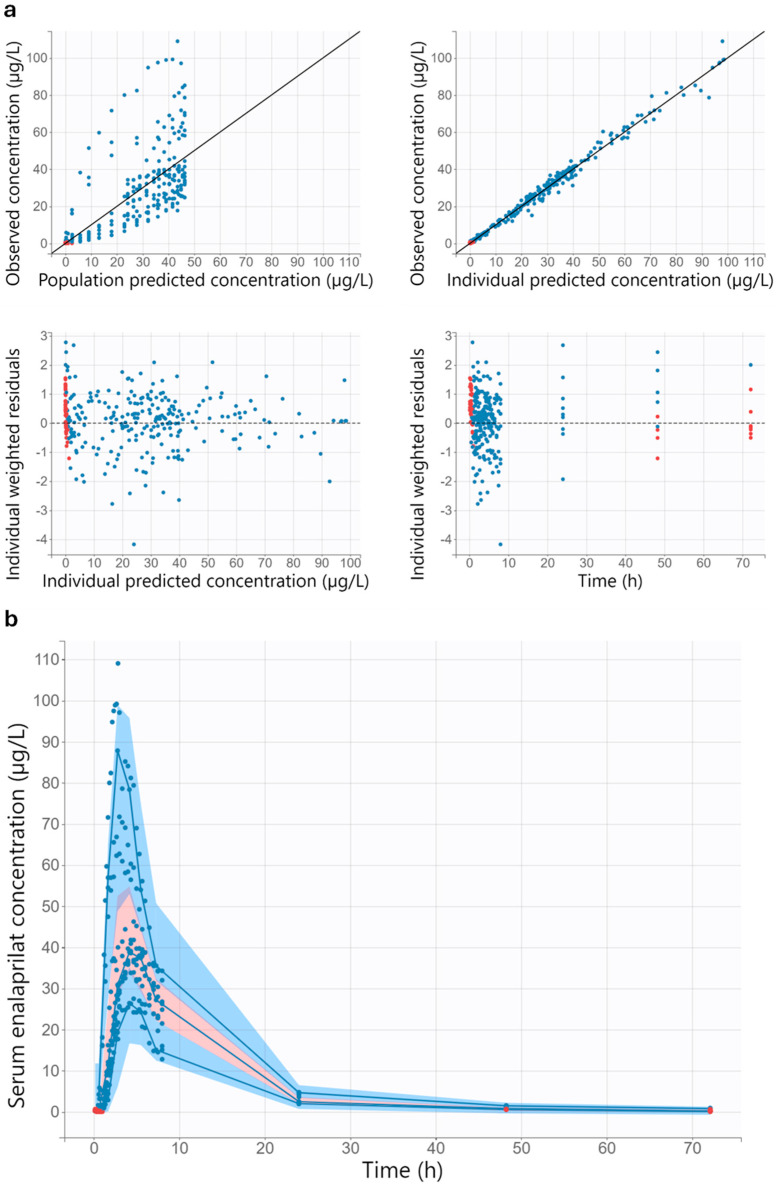
Goodness-of-fit plots (**a**) and visual predictive check (**b**) for the pharmacokinetic part of the final pharmacokinetic/pharmacodynamic model for healthy adults. All concentrations mentioned in the figure refer to the enalaprilat concentration. In all diagrams, the red dots indicate the censored data and the blue dots indicate the observed data. In the two upper diagrams in (**a**), the solid black line is the line of unity. In the two lower diagrams in (**a**), the dashed black line represents the theoretical mean. In the visual predictive check (**b**), the solid blue lines with blue dots are the 10th, 50th, and 90th percentiles of the observed data. The shaded areas represent the 90% prediction intervals of the 10th (blue), 50th (pink), and 90th (blue) percentiles of the simulated data. The purple areas are the areas where the 90% prediction interval of the 50th percentile of the simulated data overlaps with the 90% prediction interval of the 10th or 90th percentile of the simulated data.

**Figure 4:**
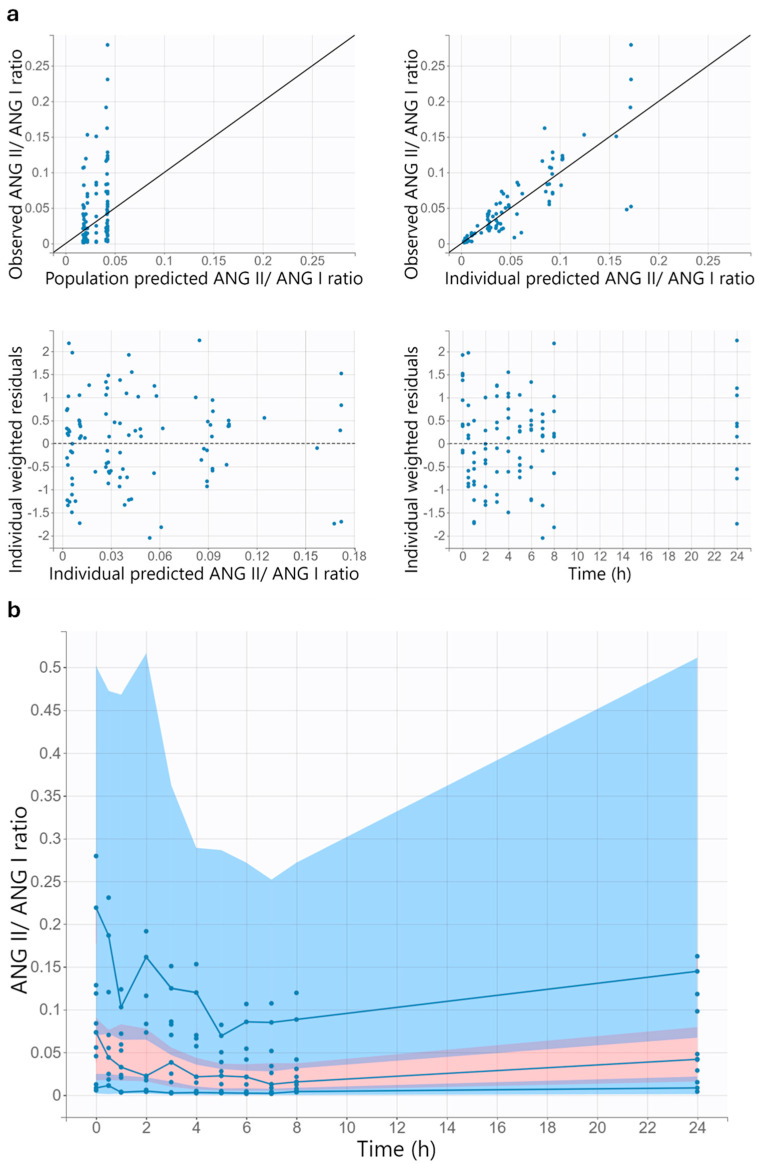
Goodness-of-fit plots for the pharmacodynamic part of the final pharmacokinetic/pharmacodynamic model for healthy adults (**a**) and visual predictive check of the final pharmacokinetic/pharmacodynamic model for healthy adults (**b**). In all diagrams, the blue dots indicate the observed data. In the two upper diagrams in (**a**), the solid black line is the line of unity. In the two lower diagrams in (**a**), the dashed black line represents the theoretical mean. In the visual predictive check (**b**), the solid blue lines with blue dots are the 10th, 50th, and 90th percentiles of the observed data. The shaded areas represent the 90% prediction intervals of the 10th (blue), 50th (pink), and 90th (blue) percentiles of the simulated data. The purple areas are the areas where the 90% prediction interval of the 50th percentile of the simulated data overlaps with the 90% prediction interval of the 10th or 90th percentile of the simulated data. ANG I, angiotensin I; ANG II, angiotensin II.

Visual inspection of the goodness-of-fit plots suggests a good model performance of the final pharmacokinetic/pharmacodynamic model for healthy adults (Figure 3 and Figure 4). A uniform distribution around the unity line is evident in the diagrams in which the observations are plotted against the individual predictions. The scatter around the unity line is lower for pharmacokinetic than for pharmacodynamic. The individual weighted residuals versus the individual predictions and versus time are evenly distributed around zero. Furthermore, most of the individual weighted residuals lie between −2 and 2.

The visual predictive checks indicate that the pharmacokinetic/pharmacodynamic model adequately describes the data (Figure 3 and Figure 4). In the relevant time range up to 24 h after administration of enalapril, the 10th, 50th and 90th percentiles of the observed data are within the corresponding 90% prediction interval. A slight underprediction of late enalaprilat concentrations was observed, as the 10th percentile of observed enalaprilat serum concentrations after 36 h is slightly above the corresponding 90% prediction interval (Figure S1).

### 3.4. Comparison of the Time Course of the Effect in Healthy Adults and Children with Heart Failure

The healthy adults received a median dose of enalapril maleate of 0.27 mg/kg (range 0.23–0.43 mg/kg), and the highest measured enalaprilat concentration was 109 µg/L. In contrast, the children with heart failure received a median first dose of enalapril maleate of 0.06 mg/kg (range 0.03–0.08 mg/kg). With repeated administration of enalapril orodispersible minitablets, the dosing interval varied between subjects. One subject received a dose of 0.06 mg/kg enalapril maleate once daily. The other subjects received enalapril orodispersible minitablets twice daily at a median daily dose of enalapril maleate of 0.11 mg/kg (range 0.06–0.27 mg/kg). The highest measured enalaprilat concentration in children with heart failure was 18.3 µg/L.

**Figure 5:**
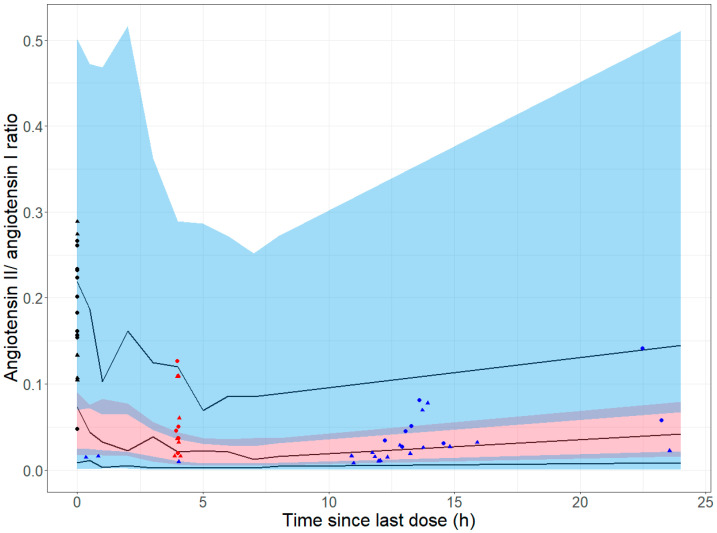
Comparison of the time course of the angiotensin II/angiotensin I ratio after enalapril administration in healthy adults and children with heart failure. The solid black lines are the 10th, 50th, and 90th percentiles of the observed data in healthy adults. The shaded areas represent the 90% prediction interval of the 10th (blue), 50th (pink), and 90th (blue) percentiles of the simulated data. The purple areas are the areas where the 90% prediction interval of the 50th percentile of the simulated data overlaps with the 90% prediction interval of the 10th or 90th percentile of the simulated data. The simulated data originate from 500 Monte Carlo simulations performed with the final pharmacokinetic/pharmacodynamic model of healthy adults. The symbols indicate the observed data in children with heart failure. The black symbols represent observations prior to the first dose of enalapril, the red symbols represent observations after the first dose of enalapril, and the blue symbols represent observations after later doses of enalapril. Observations in which the angiotensin II value was within the calibration range are shown as circles. Observations in which the angiotensin II value was below the lower limit of quantification (LLOQ) and was replaced by LLOQ/2 are shown as triangles.

Although the dosage was lower, children with heart failure achieved a similar angiotensin II/angiotensin I ratio after administration of enalapril as healthy adults (Figure 5). The time course of the angiotensin II/angiotensin I ratio after administration of enalapril is broadly similar in children with heart failure and in healthy adults. Most angiotensin II/angiotensin I ratios prior to the first dose of enalapril in children with heart failure are within the 90% prediction interval of the 90th percentile of the simulated data. This suggests that the angiotensin II/angiotensin I ratio is slightly higher in children with heart failure prior to the first dose of enalapril than in healthy adults.

### 3.5. Change in the Angiotensin II/Angiotensin I Ratio After Initial Enalapril Dose in Children with Heart Failure

For eight children (median age: 0.22 years, age range: 0.07–0.58 years) with heart failure, an angiotensin II/angiotensin I ratio was available before the first dose of enalapril and four hours afterwards. Four hours after the first dose of enalapril (median: 0.06 mg/kg enalapril maleate, range: 0.04–0.08 mg/kg enalapril maleate), the angiotensin II/angiotensin I ratio in these subjects decreased by a median of 79.3% (range: 21.4–92.7%).

### 3.6. Population Pharmacodynamic Modelling for Children with Heart Failure

#### 3.6.1. Population Pharmacodynamic Model

An I_max_ model with full inhibition was selected as a pharmacodynamic model for children with heart failure. The addition of a sigmoidicity factor was omitted because the models with and without the sigmoidicity factor barely differed in terms of the predefined model selection criteria. A proportional error model was used to describe the residual variability. The interindividual variability for the baseline effect was removed because the data were not sufficient to estimate it with an acceptable relative standard error. For the population of children with heart failure, the baseline effect was estimated to be 0.19 and the half-maximal inhibitory concentration to be 1.19 µg/L. Further parameter estimates of the final pharmacodynamic model for children with heart failure can be found in Table 3.

#### 3.6.2. Model Evaluation

**Figure 6:**
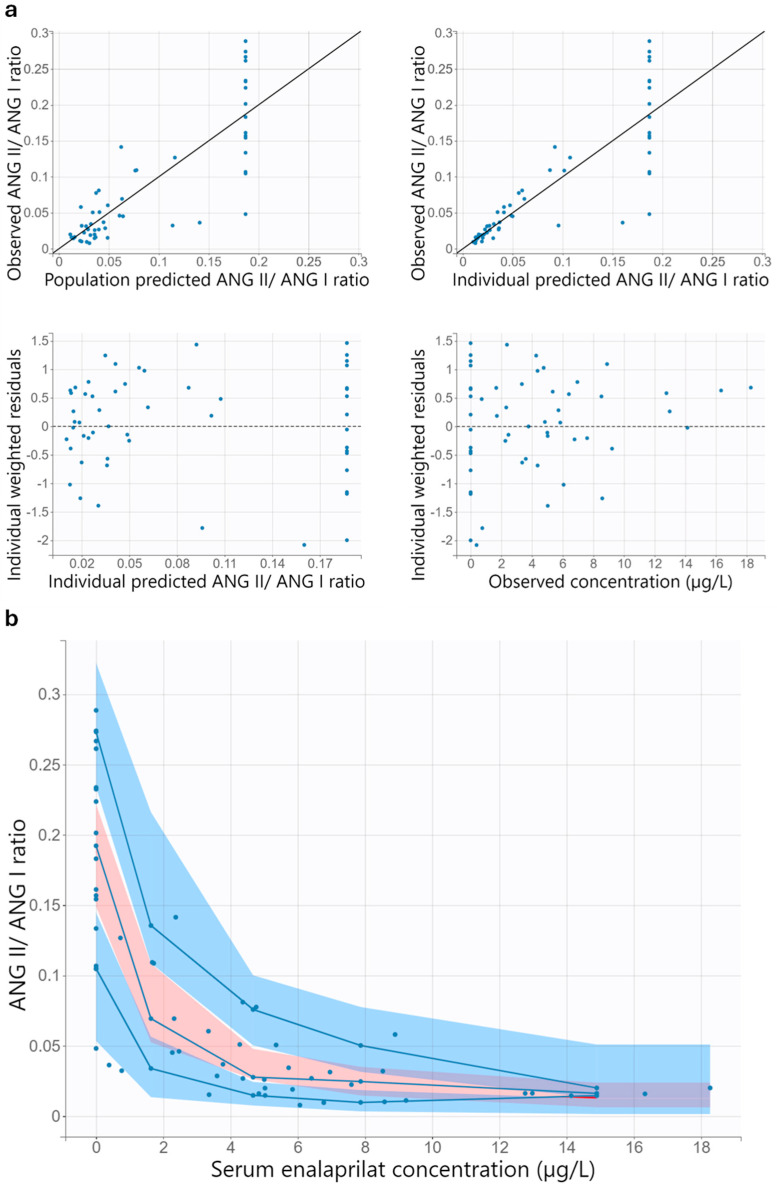
Goodness-of-fit plots (**a**) and visual predictive check (**b**) for the final pharmacodynamic model for the children with heart failure. All concentrations mentioned in the figure refer to the enalaprilat concentration. In all diagrams, the blue dots indicate the observed data. In the two upper diagrams in (**a**), the solid black line is the line of unity. In the two lower diagrams in (**a**), the dashed black line represents the theoretical mean. In the visual predictive check (**b**), the solid blue lines with blue dots are the 10th, 50th, and 90th percentiles of the observed data. The shaded areas represent the 90% prediction intervals of the 10th (blue), 50th (pink), and 90th (blue) percentiles of the simulated data. The purple areas are the areas where the 90% prediction interval of the 50th percentile of the simulated data overlaps with the 90% prediction interval of the 10th or 90th percentile of the simulated data. Red areas indicate the areas where the empirical percentile lies outside the prediction interval. ANG I, angiotensin I; ANG II, angiotensin II.

The goodness-of-fit plots of the final pharmacodynamic model suggest acceptable model performance (Figure 6). Since the final model does not include interindividual variability for the baseline effect, the model predicted the same angiotensin II/angiotensin I ratio at baseline for all subjects. Almost all individual weighted residuals lie between −2 and 2.

At enalaprilat concentrations above 14 µg/L, the visual predictive check suggests a slight underprediction of the angiotensin II/angiotensin I ratio, as the 10th percentile of the observed angiotensin II/angiotensin I ratio is slightly above the corresponding 90% prediction interval (Figure 6). Apart from this, the empirical percentiles lie within the corresponding 90% prediction interval, indicating that the pharmacodynamic model adequately describes the data.

## 4. Discussion

The angiotensin II/angiotensin I ratio was a suitable pharmacodynamic parameter for model-dependent population analyses in healthy adults and children with heart failure who have been administered enalapril. The effect of the active metabolite enalaprilat on the angiotensin II/angiotensin I ratio was adequately described by the pharmacokinetic/pharmacodynamic model developed for healthy adults and the pharmacodynamic model developed for children with heart failure. The first finding was that the population estimate for the angiotensin II/angiotensin I ratio prior to the first administration of enalapril was higher in children with heart failure than in healthy adults. Secondly, based on the change in the angiotensin II/angiotensin I ratio, it can be assumed that effective ACE inhibition was achieved with the administered dose in children with heart failure. Thirdly, the population estimate for the half-maximal inhibitory concentration was lower in children with heart failure than in healthy adults.

For the pharmacokinetic part of the pharmacokinetic/pharmacodynamic model for healthy adults, a two-compartment model with transit compartments for enalaprilat was most appropriate. The two-compartment model with a central compartment and a peripheral compartment captured the observed biphasic exponential decline in enalaprilat concentration. In the two-compartment model, the initial rapid decline in concentration is attributed to distribution into the peripheral compartment, while the subsequent slower decline is attributed to back diffusion from the peripheral compartment. A two-compartment model was also used for enalaprilat in a previously developed combined model for enalapril and enalaprilat in healthy adults [32]. Since the focus of this study was on the relationship between enalaprilat and the angiotensin II/angiotensin ratio, inactive enalapril was not included in the model. Kechagia et al. [21] also considered only the active metabolite enalaprilat for the pharmacokinetic part of their pharmacokinetic/pharmacodynamic model and likewise selected a two-compartment model. By extrapolating the estimated parameters for enalaprilat in children from this model to an adult weight of 70 kg, the apparent clearance of enalaprilat would be 18.2 L/h, and the apparent central volume of distribution of enalaprilat would be 168.8 L. The parameter estimates are thus lower than the apparent clearance of enalaprilat of 36.4 L/h and the apparent central volume of distribution of enalaprilat of 223.7 L from this study. One reason for the difference could be that the pharmacokinetic/pharmacodynamic model of Kechagia et al. [21] is based on literature data of age groups and not of individuals.

An I_max_ model with full inhibition and sigmoidicity factor for healthy adults and without sigmoidicity factor for children with heart failure was appropriate to describe the relationship between the angiotensin II/angiotensin I ratio and enalaprilat. Due to the observed time delay between measured enalaprilat concentration and effect in healthy adults, an effect compartment was included. To the authors’ knowledge, no model-dependent analyses of the angiotensin II/angiotensin I ratio as a measure of in vivo ACE activity are available for comparison. However, there are several model-dependent analyses based on in vitro ACE activity. For the relationship between enalaprilat and the inhibition of ACE activity in adults, Ribeiro et al. [33], Donnelly et al. [14], and Zapater et al. [17] used a maximum effect (E_max_) model without a sigmoidicity factor, while Ajayi et al. [16] and Hockings et al. [13] used an E_max_ model with a sigmoidicity factor. The sigmoidicity factor, also known as the Hill coefficient, influences the steepness of the concentration-effect curve. The reason that E_max_ models were used instead of I_max_ models is that the inhibition of ACE activity and not the ACE activity itself was investigated. Only Donnelly et al. [14], who investigated both the inhibition of ACE activity and blood pressure reduction as pharmacodynamic parameters, also used an effect compartment. One reason for an increasing effect over time, as observed in this study for the angiotensin II/angiotensin I ratio and by Donnelly et al. [14] for blood pressure reduction, could be that an indirect physiological response is triggered [34]. Although the reduction in angiotensin II is a direct consequence of ACE inhibition, the increase in angiotensin I results indirectly from the absence of negative feedback from angiotensin II on renin secretion and could therefore be a reason for the hysteresis observed [35].

Comparison of the adult model predictions with paediatric data and population analyses showed that the angiotensin II/angiotensin I ratio is higher in children with heart failure than in healthy adults prior to the first administration of enalapril. The review of the raw data revealed that the higher angiotensin II/angiotensin I ratio in the children with heart failure studied was mainly due to the higher angiotensin II levels compared to healthy adults. The higher angiotensin II levels in children with heart failure could be partly age-related, as a study has shown that normal children have significantly higher angiotensin II levels than adults [36]. Another study found no significant difference in angiotensin II levels between healthy children and healthy adults, but this could also be due to the fact that the samples were taken from children in a supine position and from adults in an upright position [37]. In addition to age, the higher angiotensin II levels in children with heart failure could also be due to the disease. A compensatory mechanism in paediatric heart failure is the activation of the renin–angiotensin–aldosterone system, which leads to an increase in angiotensin II [12].

Four hours after the first administration of enalapril, a median percentage reduction in the angiotensin II/angiotensin I ratio of 79.3% was achieved, suggesting effective ACE inhibition at the median initial dose of 0.06 mg/kg enalapril maleate. In eight infants with congestive heart failure, a similar mean inhibition of ACE activity of 75.5% was observed after four hours, but after a single dose of 0.25 mg/kg enalapril maleate [38]. One reason for the higher dose required could be that the congestive heart failure was described as poorly controlled with digoxin and diuretics. Secondly, the pharmacodynamic parameter examined was not exactly the same. Thirdly, extemporaneous formulations of the 5 mg tablet for adults were used, which, in contrast to the orodispersible minitablets used in the present study, carry a higher risk that parts of the dose will not be swallowed by the infant.

For the pharmacodynamic parameter angiotensin II/angiotensin I ratio, the population estimate for the half-maximal inhibitory concentration in healthy adults was 30.01 µg/L, whereas in children with heart failure it was only 1.19 µg/L. This suggests that children with heart failure may be more sensitive to enalaprilat than healthy adults. Since the change in the angiotensin II/angiotensin I ratio results from ACE inhibition, a different binding affinity of enalaprilat to ACE in children with heart failure could be a possible reason. Molecular experiments are required to investigate this assumption. Another explanation could be that more unbound enalaprilat is available for ACE inhibition in children with heart failure than in healthy adults. It is known that the amount of plasma proteins is lower in newborns and young infants [39]. Therefore, it is to be expected that the unbound fraction of enalaprilat is higher at this age.

Although the population analyses have yielded new insights, there are also limitations. Firstly, despite the relatively high interindividual variability of pharmacodynamic parameters in healthy adults, no covariates could be identified. Reasons for this could be that the number of subjects was not large enough or that the covariates examined were too similar among the subjects. Since the population analysis in healthy adults served to provide a comparison for children with heart failure, explaining pharmacodynamic variability in healthy adults was not the main objective. Secondly, the proportion of angiotensin II values below the LLOQ was relatively high in children with heart failure. However, this is not surprising, as the production of angiotensin II is inhibited by enalaprilat. The relatively high proportion of angiotensin II values below the LLOQ therefore also indicates effective ACE inhibition. To avoid excluding measurements with angiotensin II below the LLOQ, angiotensin II values below the LLOQ were replaced by LLOQ/2 for the calculation of the angiotensin II/angiotensin I ratio. Thirdly, a different analytical method was used to determine angiotensin I and angiotensin II in healthy adults and children with heart failure. However, all analysis methods used were validated at least for intra- and inter-run accuracy and precision. Differences in the measured angiotensin I and angiotensin II values due to different analysis methods should therefore be minimal. Fourthly, the pharmacodynamic model for the children with heart failure slightly underpredicted the angiotensin II/angiotensin I ratio at enalaprilat concentrations above 14 µg/L. This is probably due to the limited data available in this area. The number of samples that could be evaluated was limited by the fact that the determination of angiotensin peptides using liquid chromatography and high-resolution mass spectrometry was an additional analytical determination that was only performed for some of the pharmacodynamic samples from the LENA studies. However, in the LENA studies, more than 90% of all measured enalaprilat concentrations in ACE inhibitor-naïve subjects were below 14 µg/L. For the concentration range in which most measured enalaprilat concentrations lie, the pharmacodynamic model therefore adequately describes the data.

## 5. Conclusions

The analyses of the angiotensin II/angiotensin I ratio indicate that effective ACE inhibition and thus the prerequisite for clinical effects were achieved in children with heart failure at the given dosage. However, further studies on the effects of enalapril on clinical endpoints are necessary. The differences between children with heart failure and healthy adults identified in population analyses suggest that children with heart failure may be more sensitive to enalaprilat than healthy adults. The pharmacodynamic model developed for children with heart failure could be used in conjunction with the existing population pharmacokinetic model for enalapril and enalaprilat to simulate the effect of a given dose of enalapril on the angiotensin II/angiotensin I ratio.

## Figures and Tables

**Table 2:** Parameter estimates of the final pharmacokinetic/pharmacodynamic model for healthy adults.

Parameter	Unit	Estimate	Relative Standard Error (%)
k_tr_	h^−1^	5.31	25.7
Mtt	h	1.46	11.7
k_a_	h^−1^	1.19	8.6
CL/F	L/h	36.39	10.2
V_1_/F	L	223.71	15.3
Q/F	L/h	6.38	13.7
V_2_/F	L	108.26	27.2
k_e0_	h^−1^	0.48	21.9
γ	-	2.02	14.9
E_0_	-	0.043	39.1
IC_50_	µg/L	30.01	27.8
Interindividual variability			
IIV k_tr_	CV%	72.89	25.6
IIV Mtt	CV%	33.7	24.1
IIV CL/F	CV%	30.17	25.2
IIV V_1_/F	CV%	46.61	23.6
IIV E_0_	CV%	141.9	24.0
IIV IC_50_	CV%	79.35	30.4
Correlations			
Correlation V_1_/F, CL/F	-	0.84	12.5
Residual variability pharmacokinetic model			
Proportional error	-	0.072	12.2
Additive error	µg/L	0.42	8.7
Residual variability pharmacodynamic model			
Proportional error	-	0.41	8.9

CL/F, apparent clearance of enalaprilat; CV, coefficient of variation; E_0_, baseline effect; γ, sigmoidicity factor; IC_50_, half-maximal inhibitory concentration; IIV, interindividual variability; k_a_, absorption rate constant; k_e0_, effect compartment transfer rate constant; k_tr_, transit rate constant; Mtt, Mean transit time; Q/F, apparent intercompartmental clearance of enalaprilat; V_1_/F, apparent central volume of distribution of enalaprilat; V_2_/F, apparent peripheral volume of distribution of enalaprilat.

**Table 3:** Parameter estimates of the final pharmacodynamic model for children with heart failure.

Parameter	Unit	Estimate	Relative Standard Error (%)
E_0_	-	0.19	8.2
IC_50_	µg/L	1.19	17.9
Interindividual variability			
IIV IC_50_	CV%	59.92	24.6
Residual variability			
Proportional error	-	0.37	14.2

CV, coefficient of variation; E_0_, baseline effect; IC_50_, half-maximal inhibitory concentration; IIV, interindividual variability.

## Data Availability

Adult study: The raw data supporting the conclusions of this article will be made available by the authors upon reasonable request. Paediatric studies: The datasets from the LENA project presented in this article are not readily available due to the data protection contract of the product owner.

## Supplementary Materials

The following supporting information can be downloaded at: https://www.mdpi.com/article/10.3390/pharmaceutics17101345/s1, Table S1: Exclusion criteria of the pharmacokinetic bridging studies in subjects with and without prior treatment with angiotensin-converting enzyme inhibitors. Table S2: Dosing regimen for enalapril orodispersible minitablets. Table S3: Predefined time points of the study visits for the pharmacokinetic bridging studies and the safety follow-up study. Figure S1. Visual predictive check for the pharmacokinetic part of the final pharmacokinetic/pharmacodynamic model for healthy adults in the period from 24 to 72 h after administration of enalapril.

## Abbreviations

The following abbreviations are used in this manuscript:

ACE	Angiotensin-converting enzyme
ANG I	Angiotensin I
ANG II	Angiotensin II
AT_1_ receptor	Angiotensin II type 1 receptor
CL/F	Apparent clearance of enalaprilat
COSSAC	Conditional sampling use for stepwise approach based on correlation tests
CV	Coefficient of variation
E_0_	Baseline effect
EDTA	Ethylenediaminetetraacetic acid
E_max_	Maximum effect
γ	Sigmoidicity factor
IC_50_	Half-maximal inhibitory concentration
IIV	Interindividual variability
I_max_	Maximum inhibition
k_a_	Absorption rate constant
k_e0_	Effect compartment transfer rate constant
k_tr_	Transit rate constant
LENA	Labeling of Enalapril from Neonates up to Adolescents
LLOQ	Lower limit of quantification
Mtt	Mean transit time
NT-proBNP	N-terminal pro-B-type natriuretic peptide
Q/F	Apparent intercompartmental clearance
SD	Standard deviation
V_1_/F	Apparent central volume of distribution
V_2_/F	Apparent peripheral volume of distribution
Operational Definitions	
Phase II/III study	Seamless study design that combines the objectives of classical phase II and phase III studies to reduce the time required and the number of subjects.
Pharmacokinetic bridging study	Pharmacokinetic study with the objective of determining a dose that achieves similar exposure in children as in adults. Proof of efficacy is waived, as it is assumed that the exposure–effect relationship is similar in adults and children.
