# Supplementary material for: Angiotensin II/Angiotensin I Ratio as a New Pharmacodynamic Parameter for Population Modelling in Healthy Adults and Children with Heart Failure Treated with Enalapril
Source: Pharmaceutics. 2025 Oct 18;17(10):1345. doi: 10.3390/pharmaceutics17101345 (PMC12567347; doi:10.3390/pharmaceutics17101345)
Supplement: Supplementary file 1 [file pharmaceutics-17-01345-s001.zip › pharmaceutics-3877266-supplementary.pdf]

Supplementary Material

Angiotensin II/Angiotensin I Ratio as a New Pharmacodynamic Parameter for Population Modelling in Healthy Adults and Children with Heart Failure Treated with Enalapril

Melina Steichert [1*], Willi Cawello [1], Bjoern B. Burckhardt [1,†], Fabian K. Suessenbach [1,‡], and Stephanie Laeer [1] on behalf of the LENA Consortium

[1] Institute of Clinical Pharmacy and Pharmacotherapy, Heinrich Heine University Düsseldorf, 40225 Düsseldorf, Germany
[†] Current address: Individualized Pharmacotherapy, Institute of Pharmaceutical and Medicinal Chemistry, University of Münster, 48149 Münster, Germany
[‡] Current address: Pharmaceuticals, Research & Development, Bayer AG, 42117 Wuppertal, Germany

[*]**Correspondence:** melina.steichert@hhu.de

Journal: Pharmaceutics

Table S1. Exclusion criteria of the pharmacokinetic bridging studies in subjects with and without prior treatment with angiotensin-converting enzyme inhibitors.

Exclusion criteria
• Severe heart failure and/or end stage heart failure precluding introduction or continuation of ACE inhibitor
• Too low blood pressure, e.g. less than P5 for age
• Restrictive and hypertrophic cardiomyopathies
• Obstructive valvular disease (peak echocardiographic gradient more than 30 mmHg)
• Uncorrected severe peripheral stenosis of large arteries including severe coarctation of the aorta
• Severe renal impairment with serum creatinine above two times the upper limit of normal according to the hospital's test methodology
• History of angioedema
• Hypersensitivity to ACE inhibitor
• Concomitant medication: Dual ACE inhibitor therapy, Renin inhibitors, Angiotensin II antagonists or non-steroidal anti-inflammatory drugs except acetylsalicylic acid only for antiplatelet therapy
• Already enrolled in an interventional trial with an investigational drug, unless no interference with the current study can be shown

ACE, angiotensin-converting enzyme

Table S2. Dosing regimen for enalapril orodispersible minitablets.

Age	Dose	Type of dose	Enalapril daily dose (ODMTs)	Enalapril daily dose (mg)	Enalapril morning dose	Enalapril evening dose
1 day to below 6 months	1st	Titration dose	10% of 1 ODMT 0.25 mg[a]	0.025	10% of 1 ODMT 0.25 mg	-
(ca. 2.5 to 7 kg)	2nd	Titration dose	50% of 1 ODMT 0.25 mg[a]	0.125	50% of 1 ODMT 0.25 mg	-
	3rd	Titration dose	1 ODMT 0.25 mg	0.25	1 ODMT 0.25 mg	-
	4th	Titration dose	2 ODMT 0.25 mg	0.5	1 ODMT 0.25 mg	1 ODMT 0.25 mg
	5th	Target dose	4 ODMT 0.25 mg	1	2 ODMT 0.25 mg	2 ODMT 0.25 mg
		Maximum dose	2 ODMT 1 mg	2	1 ODMT 1 mg	1 ODMT 1 mg
6 months to below 3 years	1st	Titration dose	1 ODMT 0.25 mg	0.25	1 ODMT 0.25 mg	-
(ca. 8 to 15 kg)	2nd	Titration dose	2 ODMT 0.25 mg	0.5	1 ODMT 0.25 mg	1 ODMT 0.25 mg
	3rd	Titration dose	4 ODMT 0.25 mg	1	2 ODMT 0.25 mg	2 ODMT 0.25 mg
	4th	Target dose	2 ODMT 1 mg	2	1 ODMT 1 mg	1 ODMT 1 mg
		Maximum dose	4 ODMT 1 mg	4	2 ODMT 1 mg	2 ODMT 1 mg
3 to below 8 years	1st	Titration dose	2 ODMT 0.25 mg	0.5	1 ODMT 0.25 mg	1 ODMT 0.25 mg
(ca. 16 to 25 kg)	2nd	Titration dose	4 ODMT 0.25 mg	1	2 ODMT 0.25 mg	2 ODMT 0.25 mg
	3rd	Titration dose	2 ODMT 1 mg	2	1 ODMT 1 mg	1 ODMT 1 mg
	4th	Target dose	4 ODMT 1 mg	4	2 ODMT 1 mg	2 ODMT 1 mg
		Maximum dose	8 ODMT 1 mg	8	4 ODMT 1 mg	4 ODMT 1 mg
8 to below 12 years	1st	Titration dose	4 ODMT 0.25 mg	1	2 ODMT 0.25 mg	2 ODMT 0.25 mg
(ca. 26 to 40 kg)	2nd	Titration dose	2 ODMT 1 mg	2	1 ODMT 1 mg	1 ODMT 1 mg
	3rd	Titration dose	4 ODMT 1 mg	4	2 ODMT 1 mg	2 ODMT 1 mg
	4th	Target dose	8 ODMT 1 mg	8	4 ODMT 1 mg	4 ODMT 1 mg
		Maximum dose	16 ODMT 1 mg	16	8 ODMT 1 mg	8 ODMT 1 mg

ODMT, orodispersible minitablets

[a] Option for very young and low weight patients in whom the investigator considers an initial dose of 1 x 0.25 mg enalapril ODMT to be too high

Table S3. Predefined time points of the study visits for the pharmacokinetic bridging studies and the safety follow-up study.

Visit	Time point
Screening Visit[a]	Day -21 to Day -1
Initial Dose Visit	Day 0
Titration Visits[b]	Day 2-Day x<br>(Visit Window 2-7 days from the previous visit)
Dose Confirmation Visit[c]	Day 3 to Day 8 from last Titration Visit
First Study Control Visit	Day 14 ± 2d
Second Study Control Visit	Day 28 ± 2d
Third Study Control Visit	Day 42 ± 2d
End-of-Study Visit of the pharmacokinetic bridging studies/ First Follow-up Study Visit[d]	Day 56 ± 2d
Second Follow-up Study Visit	Month 3 ± 7d
Third Follow-up Study Visit	Month 6 ± 7d
Fourth Follow-up Study Visit	Month 9 ± 7d
Follow-up Study Close-out Visit	Month 12 ± 7d

[a] Screening Visit and Initial Dose Visit could be combined in one visit, if the children had a weight of more than 4.2 kg.

[b] Number of Titration Visits was dependent on the age of the patient and the judgement of the investigator. For infants from 1 to 6 months a maximum of 4 titration visits were planned and for all other children older than 6 months a maximum of 3 titration visits were planned.

[c] Dose Confirmation Visit and First Study Control Visit could be combined in one visit.

[d] The First Follow-up Study Visit was, de facto, the End-of-Study Visit of the respective pharmacokinetic bridging study.

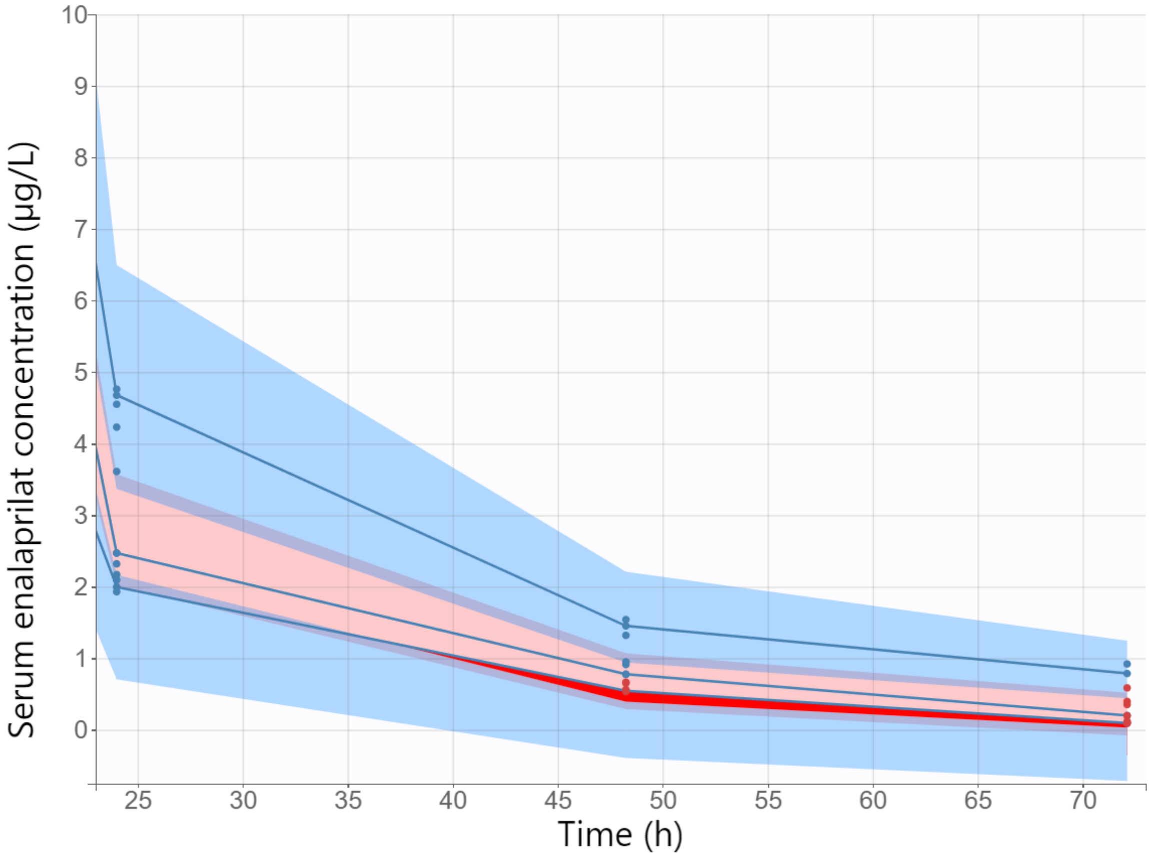

Figure S1. Visual predictive check for the pharmacokinetic part of the final pharmacokinetic/pharmacodynamic model for healthy adults in the period from 24 to 72 hours after administration of enalapril. The red dots indicate the censored data, and the blue dots indicate the observed data. The solid blue lines with blue dots are the 10th, 50th, and 90th percentiles of the observed data. The shaded areas represent the 90% prediction intervals of the 10th (blue), 50th (pink), and 90th (blue) percentiles of the simulated data. The purple areas are the areas where the 90% prediction interval of the 50th percentile of the simulated data overlaps with the 90% prediction interval of the 10th or 90th percentile of the simulated data. Red areas indicate the areas where the empirical percentile lies outside the prediction interval.
